# Lysosomal storage diseases in the era of COVID-19: a report of an Egyptian case of alpha-fucosidosis and a summary of the lysosomal storage diseases-COVID-19 relationship

**DOI:** 10.1186/s43042-022-00350-5

**Published:** 2022-09-15

**Authors:** Heba Saed El-Amawy, Heba Dawoud

**Affiliations:** Faculty of Medicine, Tanta University, Tanta, Egypt

**Keywords:** Alpha-fucosidosis, Lysosomal storage diseases, COVID-19, Respiratory infection, Angiokeratoma

## Abstract

**Background:**

We present a case of alpha-fucosidosis, a lysosomal storage disorder, from Egypt. The report also includes a brief review of the COVID-19 and lysosomal storage diseases relationship.

**Case presentation:**

A female patient aged 18 years, diagnosed with type II fucosidosis, based on the cutaneous signs, characteristic facies, and systemic symptoms, and diagnosis was confirmed using genetic analysis. The patient died from COVID-19 pneumonia during the COVID-19 pandemic after getting the infection from her father and being hospitalized.

**Conclusions:**

Patients with lysosomal storage diseases with local or systemic immune suppression may be predisposed to respiratory complications of COVID-19. Intense care with protective guidelines should be applied to those patients.

## Background

Alpha-fucosidosis is an autosomal recessive (AR) lysosomal storage disorder (LSD) characterized by accumulation of fucose-rich glycoproteins and glycolipids in different organs, and a wide range of clinical symptoms. The COVID-19 pandemic is still evolving and is proved to have a significant effect on a variety of health issues. We present an 18-year-old female who was diagnosed with α-fucosidosis. Shortly after diagnosis, the patient died from COVID-19-related pneumonia. To our knowledge, this is the first case report documenting a direct association between LSDs and development of COVD-19 life-threatening complications.

## Case presentation

**Fig. 1:**
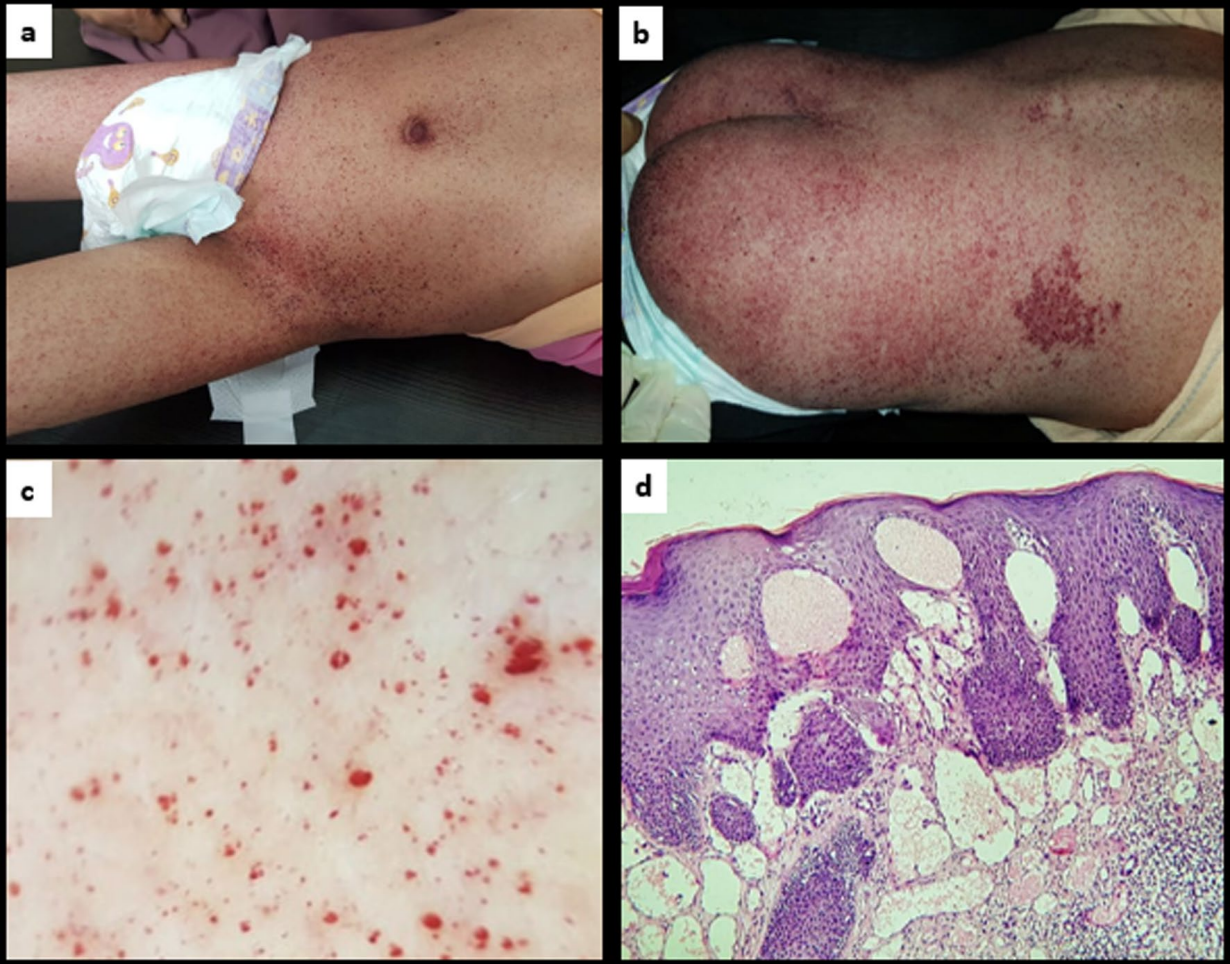
**a** Multiple minute dark erythematous angiokeratoma papules on the trunk and thigh. **b** Disseminated angiokeratoma lesions on back and buttocks. **c** Dermoscopy of the multiple angiokeratomas revealing multiple small red lacunae. **d** Histopathological examination showing vascular ectasia of the papillary dermis with the epidermis encircling the dilated vascular spaces, and overlying epidermal hyperplasia characterized by acanthosis, elongation of the rete (H and E, ×40)

A female Egyptian girl aged 18 years, at the time of presentation, was referred from the dermatology clinic to the genetics unit after being diagnosed with diffuse angiokeratomas based on clinical and dermoscopy examination, to be confirmed by histopathology (Fig. 1). Family history was positive for consanguinity, and her brother was known to have intellectual disability, short attention span, and cleft palate that was operated on. The pregnancy history was unremarkable, and the patient was born full term through a normal vaginal delivery, with no history of postnatal incubation or jaundice. Past history was positive for delayed developmental milestones starting from early age. At the age of 11 months, she was only able to sit supported, and she started to walk supported and speak few words by the age of 2 years. Global developmental delay has been noticed at the age of 3 years. When the parents sought medical advice, the patient was misdiagnosed as cerebral palsy. The manifestations became worse by time with progression of the motor delay over the years. The patient suffered recurrent attacks of pneumonia that required hospitalization. Tonsillectomy was performed at the age of 3 years due to tonsillar hypertrophy that was complicated by chronic upper airway obstruction and.

Her skin lesions began as faint erythematous telangiectatic rash at the age of 4 years that progressed gradually. Her parents gave a history of cold extremities with bluish discoloration in cold weather. There were numerous concerns by parents regarding the progressive regression of her mental and motor milestones, failure to thrive, multiple joint contractures, and widespread telangiectasia in her skin. By the age of 5 years, she lost the ability to sit or walk supported. She experienced her first attack of generalized tonic–clonic convulsions at the age of 6 years and started carbamazepine treatment. At the time of presentation, she was on double anti-epileptic drugs. By the age of 7 years, she could barely sit supported. Scoliosis was obvious at the age of 9 years. The patient had limb dystonic movements that led eventually to permanent bone deformities and joint contractures.

**Fig. 2:**
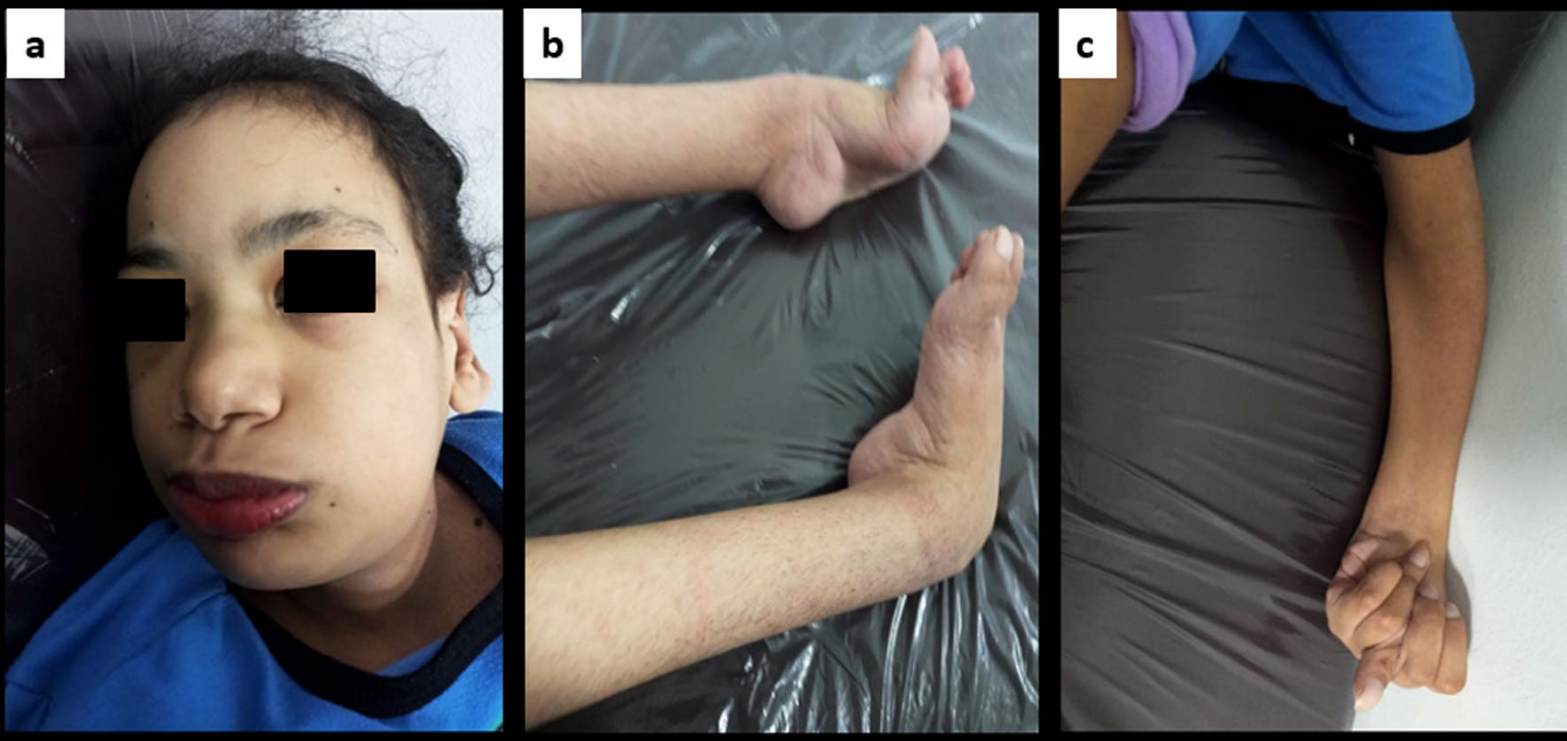
18-years-old female patient, **a** The patient’s coarse facies with right plagiocephaly, small cupped low set ear, depressed nasal bridge, triangular face, upward slanting, epicanthus fold, squint and broad eye gaps. **b** Extensive contracture at ankle, secondary to the severe lower limb spasticity. **c** Overlapped fingers due to marked Spasticity of the left hand

At the time of examination, the patient weighed 23 kg (Z-score = − 14), with a height of 122 cm (Z-score = − 6.31, below the third percentile curves for her age), and a head circumference of 53 cm (− 1 SD). She had coarse facial features (Fig. 2).

Her vitals were within normal range. Cardiac examination revealed tachycardia with soft cardiac murmur and neck pulsations. She had abdominal distension with firm palpable liver. Abdominal ultrasonography showed splenomegaly 15 cm below the costal margin and hepatomegaly 5 cm below the costal margin. Neurological and skeletal examination exhibited spasticity, exaggerated reflexes, scoliosis, and widespread joint contracture, especially at the wrist and metacarpophalangeal joints. Brain MRI and X-ray imaging were done at the first presentation and showed thinning of gray matter, dysostosis multiplex of long bones, pelvis, and spines, and scoliosis (Images are not available for publication). Based on the clinical and radiologic features, fucosidosis was suspected. Accordingly, the activity of the enzyme alpha-L-fucosidase was measured by fluorometric assay, and it was shown to be deficient (< 0.05 (LOD) μmol/L/h, normal reference is ≥ 6.3 μmol/L/h). Sequencing of FUCA1 gene including NGS-based CNV analysis revealed homozygous FUCA1, c.1100del p.(Glu367Glyfs*42) variant which creates a shift in the reading frame starting at codon 367. The new reading frame ends in a stop codon 41 positions downstream. It was classified as pathogenic (class 1), according to the recommendations of CENTOGENE and ACMG. Consequently, the genetic diagnosis of autosomal recessive fucosidosis was confirmed.

**Fig. 3:**
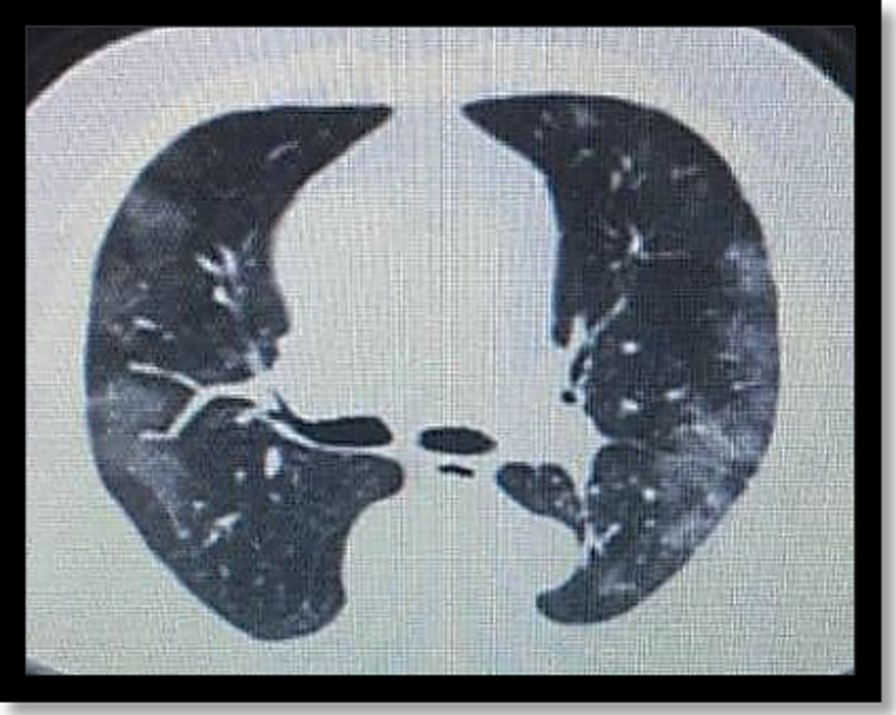
Axial nonenhanced chest CT of the patient showing bilateral patches of ground-glass opacities in a peripheral and sub-pleural distribution, diagnosing COVID-19 pneumonia

Two months after the diagnosis was made, the patient’s father was diagnosed as COVID-19 with moderate symptoms of cough and fever (38.5 °C). He recovered without complications. Few days following his diagnosis, the patient developed a high-grade fever of 40 °C that failed to respond to antipyretics, a moderate productive cough, and difficulty in breathing. Her extremities were cyanosed. Her mother reported loss of the usual body spasticity, fatigue, and flaccidity of the patient’s lower limbs. Being a COVID-19 suspect, she was admitted to the nearest isolation hospital and was diagnosed as a COVID-19-induced pneumonia based on positive PCR test and the characteristic findings in her CT chest (Fig. 3). On admission, she was confused and distressed (respiratory rate: 40 breaths/min, pulse rate: 110 beats/min, and oxygen saturation: 75%). Her peripheral blood lymphocytic count was very low (0.30 × 10^9^/L). She started on oxygen therapy through non-rebreathing face mask, anticoagulants, broad-spectrum antibiotics, and tocilizumab 400 mg in two divided doses with 12 h interval. Despite therapy, her condition deteriorated with drop of oxygen saturation to 60–65%, which required a mechanical ventilator. She died three weeks following initial diagnosis with COVID-19 infection.

## Discussion

Alpha-fucosidosis is a very rare AR lysosomal storage condition with a male-to-female ratio of approximately 1.75:1. It is linked to a mutation in the L-fucosidase 1 gene (FUCA1), which causes an extreme deficiency of the lysosomal enzyme alpha-L-fucosidase [1]. Since alpha-L-fucosidase is involved in the cleavage of fucosyl residues from whole glycoconjugates, a deficiency or absence of the enzyme in fucosidosis disorder can impair lysosomal degradation of fucose-containing glycoproteins and glycolipids, resulting in deposition of these substances in various body tissues with a myriad of symptoms [2]. Fucosidosis is divided into two sub-types based on the age of onset and life expectancy: type I which starts at 6 months of age and progresses rapidly, resulting in neurological deterioration and death during the first decade of life; and type II which begins between 12 and 24 months of age and progresses more slowly, resulting in longer survival until the second decade. However, there is no specific clinical distinction between type I and type II. Moreover, among members of the same family, there is a wide range of variation in clinical presentation [3]. Our case is more likely to be type II fucosidosis, based on the age of presentation. Less than 120 cases of fucosidosis have been identified to date, with many cases occurring in Italians and Mexican Indians in New Mexico and Colorado [2]. To our knowledge, this is the second report of fucosidosis in Egyptian patients. The first case was a 2-year-old boy who had neither coarse facial features nor cutaneous angiokeratoma at the time of diagnosis [4].

Severe global developmental delay (95%), muscle stiffness (87%), coarse facies (79%), recurrent respiratory infections (78%), visceromegaly (44%), seizures (38%), and abnormal bone development (58%) are among the extracutaneous manifestations of alpha-Fucosidosis, while the mucocutaneous features include tortuous conjunctival vessels (53%), angiokeratoma corporis diffusum (ACD) (52%), widespread telangiectasias, acrocyanosis, purple transversal distal nail bands, increased palmo‐plantar vascularity, dry thin skin, and sweating abnormalities (hyper‐ or hypo‐hidrosis) [1, 5]. Several reported patients with fucosidosis were found to have no cutaneous symptoms [3]. With age, the risk of developing ACD and the severity of the condition increase. Patients can present with telangiectasia at first, then develop angiokeratomas later [1]. The accumulation of pathologic material inside the cytoplasm of endothelial cells, which could trigger their apoptosis, with continuous reactive regeneration, leading to the emergence of newly developed ectatic capillaries, is thought to be the cause of ACD in various metabolic diseases, including fucosidosis [3]. ACD is more common in patients that have lived longer. Besides fucosidosis, ACD has also been reported in Fabry disease (FD), sialidosis, α-mannosidosis, β-mannosidosis, aspartylglucosaminuria, galactosialidosis [6].

Due to glycolipid accumulation, prominent gray matter atrophy, prominent white matter demyelination, dysostosis multiplex, kyphosis or scoliosis, widening of long bones diaphysis, and thickening of the skull are all seen on radiologic tests [2]. Recurrent pulmonary infections and neurological decline are the most common causes of death [7].

In our patient, the majority of her systems were involved. The genetic testing is the definitive way to diagnose fucosidosis, however, the radiologic findings, together with clinical signs and symptoms, can be helpful. There is currently no accepted treatment for fucosidosis, and the disorder is treated predominantly by supportive and symptomatic therapy, including muscle relaxants, physical therapy and massage for spasticity, feeding therapy, antacids and pureed food for the feeding problems and gastroesophageal reflux [8]. Enzyme replacement therapy and hematopoietic cell transplantation are still in the early stages of development [9].

Lysosomal storage diseases (LSDs) are among the disorders associated with an exceptionally high risk of serious COVID-19 infection [10]. The immune system function is impaired in several LSDs. Gaucher disease (GD), mucopolysaccharidosis (MPS VII), and -mannosidosis are LSDs that tend to suppress the immune system. Antigen presentation and processing, secretion of perforins by cytotoxic-T lymphocytes, and release of pro-inflammatory mediators by mast cells are all immune system functions in which the lysosome plays a key role [11].

In fucosidosis patients, recurrent infections of the sinus cavities, ears, and airway are thought to be caused by a local deficiency in mucus clearance rather than a systemic immune defect. The fact that recurrent infections in such patients are restricted to areas of mucus-secreting ciliated epithelia, with no evidence of a rise in the prevalence of extrapulmonary infections, supports this hypothesis. Alterations in the mucus cross-linking and viscoelasticity are caused by changes in enzymatic cleavage of sugars combined with incomplete assembly of mucus glycoprotein as well as failure of glycoprotein secretion in fucosidosis. The abnormally watery, low-elasticity mucus will be difficult to remove from the airway, contributing to chronic pulmonary infection [12]. Recurrent respiratory infections are one of the most common symptoms in patients with fucosidosis, with a prevalence of 78%. Skeletal deformities of the chest wall can be an additional risk factor for COVID-19 complications due to pulmonary and cardiac power limitation and insufficient airway ventilation. Hepatosplenomegaly can reduce lung volume and hasten respiratory decline in these patients, resulting in a fatal COVID-19 infection [10]. Regarding the present case, we suspected that her extreme hepatosplenomegaly and scoliosis contributed to her severe COVID-19 infection and death.

COVID-19 had a major effect on LSD patients in Italy, with 49 percent of patients undergoing enzyme replacement therapy in hospitals experiencing delays compared to just 6% of those treated at home, with fear of infection (62.9%) and reorganization of infusion centers (37%) being the key reasons for missed infusions. COVID-19 was not present in any of the 102 people interviewed [13]. Since LSD patients often suffer from a multisystem disorder, the study found them to be at a high risk of developing serious complications if infected with SARS-CoV-2 [13]. In a separate study from Italy, people with LSDs reported higher levels of anxiety and psychological fear, particularly, they have rare diseases that are susceptible to complications if they contract COVID-19, but there was no statistically significant difference when compared to controls [14]. Just one patient with FD had laboratory-verified COVID-19 among the approximately 400 confirmed MPS, GD, and FD patients in Morocco. He had no signs of SARS-CoV-2 infection and was kept in isolation at home for 14 days [10]. The accumulation of glycosphingolipid in FD, which modifies and shapes the biology of gut bacteria and may have a prophylactic effect against SARS-CoV-2 disease, may be the reason for asymptomatic infection in this patient despite his critical condition [15]. In a cohort study of around 550 symptomatic cases of GD from Israel and Australia (median age 46 (18–94) years), only one woman, who was pregnant in her third trimester and was 24 years old, had a reported COVID-19 infection. She had a moderate clinical course that was only handled by a 14-day quarantine [16].

Recent studies have come up with some very plausible explanations for the low incidence of LSD patients infected with COVID-19. Balout et al. hypothesized that the intracellular biochemical abnormalities of LSDs, in general, and Niemann–Pick disease type C (NPC) in particular, could establish a "unfavorable" host cell environment for SARS-CoV-2 entry, trafficking, and fusion. The suggested mechanism could be, first and foremost, altered plasma membrane and lipid raft composition in NPC, which could influence the trafficking of ACE2, the primary host cell membrane receptor responsible for viral entry. Other reasons include decreased cathepsin L activity in NPC, a key protease needed for successful SARS-CoV-2 fusion, and a high level of oxysterols in NPC, which have potent antiviral properties. The authors hypothesized that LSDs may be a successful therapeutic axis for COVID-19 in the future [17]. When exposed to COVID-19 infection, Zimran et al. hypothesized that the accumulated glycosphingolipids in patients with GD might induce immune tolerance rather than inflammation, resulting in a defensive line against infection [16]. Furthermore, LSDs with accumulating metabolites can induce new immune activation involving Natural Killer cells and other immune cells, such as B cells, resulting in different immune responses [18].

## Conclusion

Despite suggestions that LSDs may be safe from serious COVID-19, studies show that patients with lysosomal storage disease should take precautions during the COVID-19 pandemic to avoid respiratory complications, especially in LSDs with local or systemic immune suppression. Adjusting ERT doses to double the dosage for infrequent hospital visits, following all preventive measures during hospital visits, and paying attention to drug-drug interactions between the medications used to treat LSDs and COVID-19 care if the patients had COVID-19 are all potential guidelines. In these cases, early diagnosis and intervention are critical for a successful outcome and lower letter fatalities. Further studies are needed to investigate the impact of COVID-19 on the respiratory tract in patients with lysosomal storage diseases.

## Data Availability

All data generated or analyzed during this study are included in this published article.

## Abbreviations

ACD: Angiokeratoma corporis diffusum
ACE2: Angiotensin-converting enzyme 2
ACMG: American College of Medical Genetics and Genomics
AR: Autosomal recessive
CNV: Copy number variant
FD: Fabry disease
FUCA 1: α-L-fucosidase gene
GD: Gaucher disease
LOD: Limit of detection
LSD: Lysosomal storage disorder
MPS: Mucopolysaccharidosis
NGS: Next-generation sequencing
NPC: Niemann–Pick disease type C
SD: Standard deviation
SpO2: Oxygen saturation
